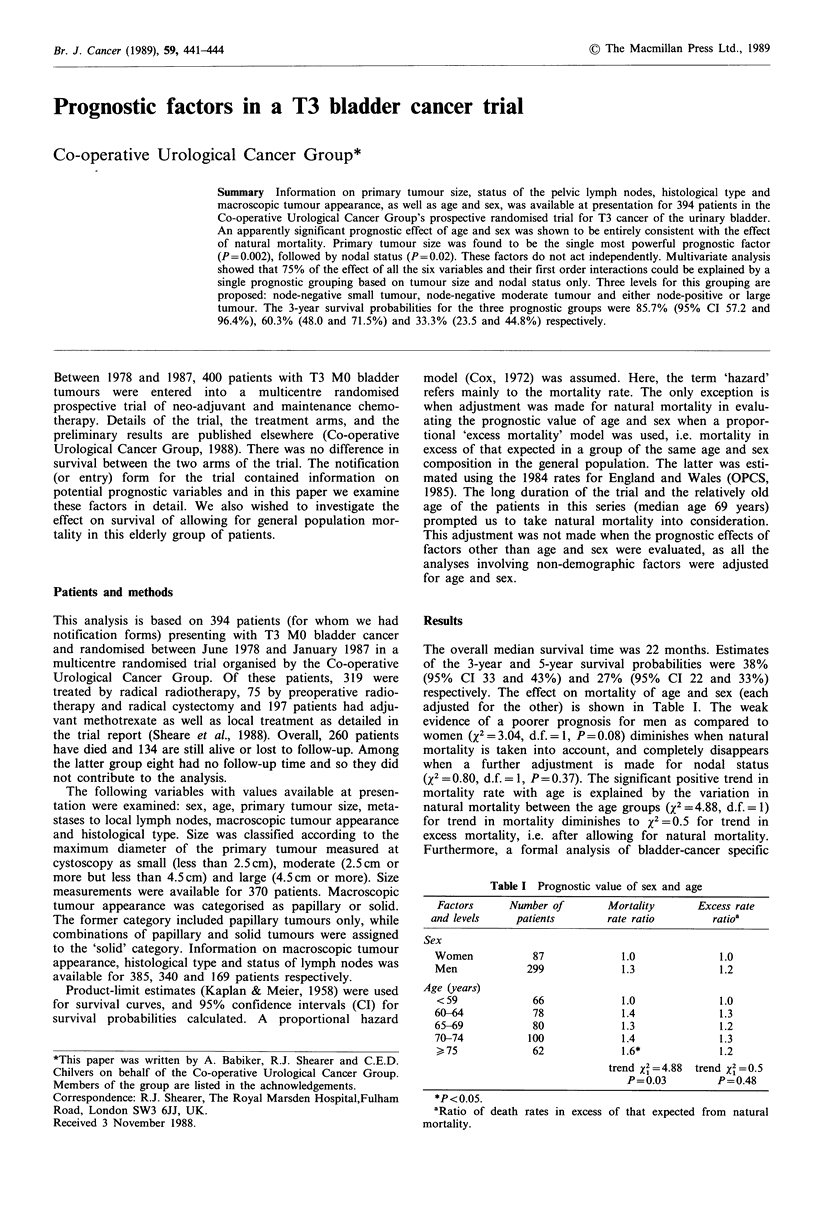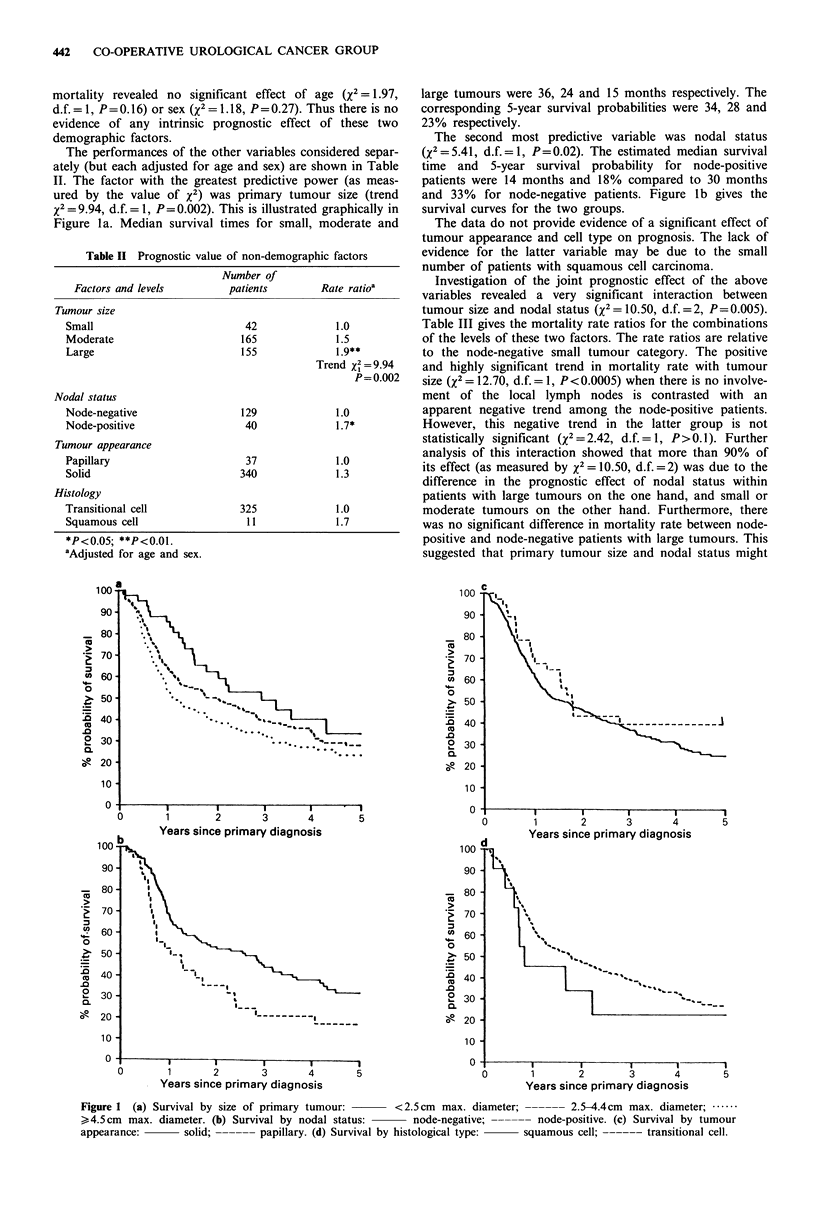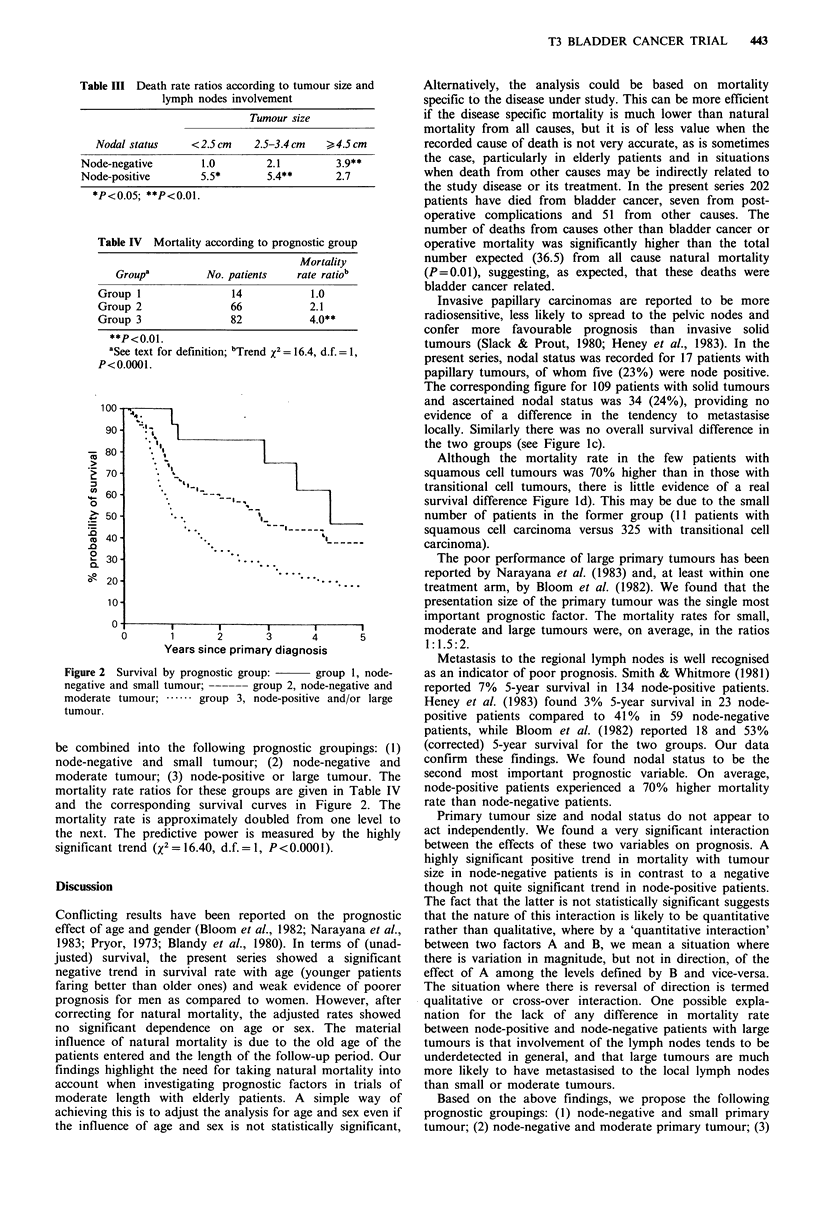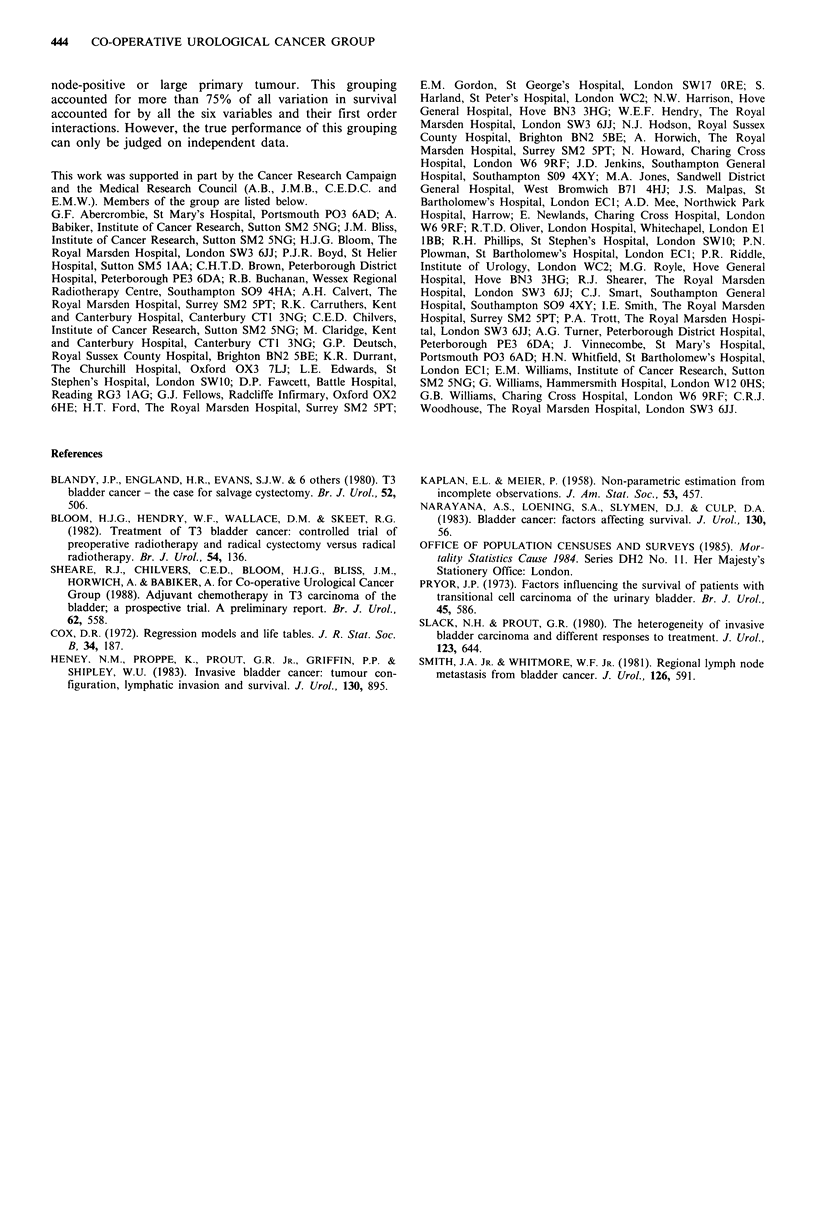# Prognostic factors in a T3 bladder cancer trial. Co-operative Urological Cancer Group.

**DOI:** 10.1038/bjc.1989.90

**Published:** 1989-03

**Authors:** A. Babiker, R. J. Shearer, C. E. Chilvers

**Affiliations:** Royal Marsden Hospital, London, UK.

## Abstract

Information on primary tumour size, status of the pelvic lymph nodes, histological type and macroscopic tumour appearance, as well as age and sex, was available at presentation for 394 patients in the Co-operative Urological Cancer Group's prospective randomised trial for T3 cancer of the urinary bladder. An apparently significant prognostic effect of age and sex was shown to be entirely consistent with the effect of natural mortality. Primary tumour size was found to be the single most powerful prognostic factor (P = 0.002), followed by nodal status (P = 0.02). These factors do not act independently. Multivariate analysis showed that 75% of the effect of all the six variables and their first order interactions could be explained by a single prognostic grouping based on tumour size and nodal status only. Three levels for this grouping are proposed: node-negative small tumour, node-negative moderate tumour and either node-positive or large tumour. The 3-year survival probabilities for the three prognostic groups were 85.7% (95% CI 57.2 and 96.4%), 60.3% (48.0 and 71.5%) and 33.3% (23.5 and 44.8%) respectively.


*Br. J. Cancer* (1989), **59**, 441–444 © The Macmillan Press Ltd., 1989

### Prognostic factors in a T3 bladder cancer trial

Co-operative Urological Cancer Group*

**Summary** Information on primary tumour size, status of the pelvic lymph nodes, histological type and macroscopic tumour appearance, as well as age and sex, was available at presentation for 394 patients in the Co-operative Urological Cancer Group's prospective randomised trial for T3 cancer of the urinary bladder. An apparently significant prognostic effect of age and sex was shown to be entirely consistent with the effect of natural mortality. Primary tumour size was found to be the single most powerful prognostic factor ($P=0.002$), followed by nodal status ($P=0.02$). These factors do not act independently. Multivariate analysis showed that 75% of the effect of all the six variables and their first order interactions could be explained by a single prognostic grouping based on tumour size and nodal status only. Three levels for this grouping are proposed: node-negative small tumour, node-negative moderate tumour and either node-positive or large tumour. The 3-year survival probabilities for the three prognostic groups were 85.7% (95% CI 57.2 and 96.4%), 60.3% (48.0 and 71.5%) and 33.3% (23.5 and 44.8%) respectively.

Between 1978 and 1987, 400 patients with T3 M0 bladder tumours were entered into a multicentre randomised prospective trial of neo-adjuvant and maintenance chemotherapy. Details of the trial, the treatment arms, and the preliminary results are published elsewhere (Co-operative Urological Cancer Group, 1988). There was no difference in survival between the two arms of the trial. The notification (or entry) form for the trial contained information on potential prognostic variables and in this paper we examine these factors in detail. We also wished to investigate the effect on survival of allowing for general population mortality in this elderly group of patients.

### Patients and methods

This analysis is based on 394 patients (for whom we had notification forms) presenting with T3 M0 bladder cancer and randomised between June 1978 and January 1987 in a multicentre randomised trial organised by the Co-operative Urological Cancer Group. Of these patients, 319 were treated by radical radiotherapy, 75 by preoperative radiotherapy and radical cystectomy and 197 patients had adjuvant methotrexate as well as local treatment as detailed in the trial report (Sheare *et al.*, 1988). Overall, 260 patients have died and 134 are still alive or lost to follow-up. Among the latter group eight had no follow-up time and so they did not contribute to the analysis.

The following variables with values available at presentation were examined: sex, age, primary tumour size, metastases to local lymph nodes, macroscopic tumour appearance and histological type. Size was classified according to the maximum diameter of the primary tumour measured at cystoscopy as small (less than 2.5 cm), moderate (2.5 cm or more but less than 4.5 cm) and large (4.5 cm or more). Size measurements were available for 370 patients. Macroscopic tumour appearance was categorised as papillary or solid. The former category included papillary tumours only, while combinations of papillary and solid tumours were assigned to the 'solid' category. Information on macroscopic tumour appearance, histological type and status of lymph nodes was available for 385, 340 and 169 patients respectively.

Product-limit estimates (Kaplan & Meier, 1958) were used for survival curves, and 95% confidence intervals (CI) for survival probabilities calculated. A proportional hazard model (Cox, 1972) was assumed. Here, the term 'hazard' refers mainly to the mortality rate. The only exception is when adjustment was made for natural mortality in evaluating the prognostic value of age and sex when a proportional 'excess mortality' model was used, i.e. mortality in excess of that expected in a group of the same age and sex composition in the general population. The latter was estimated using the 1984 rates for England and Wales (OPCS, 1985). The long duration of the trial and the relatively old age of the patients in this series (median age 69 years) prompted us to take natural mortality into consideration. This adjustment was not made when the prognostic effects of factors other than age and sex were evaluated, as all the analyses involving non-demographic factors were adjusted for age and sex.

### Results

The overall median survival time was 22 months. Estimates of the 3-year and 5-year survival probabilities were 38% (95% CI 33 and 43%) and 27% (95% CI 22 and 33%) respectively. The effect on mortality of age and sex (each adjusted for the other) is shown in Table I. The weak evidence of a poorer prognosis for men as compared to women ($\chi^2=3.04$, d.f. = 1, $P=0.08$) diminishes when natural mortality is taken into account, and completely disappears when a further adjustment is made for nodal status ($\chi^2=0.80$, d.f. = 1, $P=0.37$). The significant positive trend in mortality rate with age is explained by the variation in natural mortality between the age groups ($\chi^2=4.88$, d.f. = 1) for trend in mortality diminishes to $\chi^2=0.5$ for trend in excess mortality, i.e. after allowing for natural mortality. Furthermore, a formal analysis of bladder-cancer specific

**Table I** Prognostic value of sex and age

| *Factors and levels* | *Number of patients* | *Mortality rate ratio* | *Excess rate ratio*[a] |
|---|---|---|---|
| *Sex* | | | |
| Women | 87 | 1.0 | 1.0 |
| Men | 299 | 1.3 | 1.2 |
| *Age (years)* | | | |
| <59 | 66 | 1.0 | 1.0 |
| 60–64 | 78 | 1.4 | 1.3 |
| 65–69 | 80 | 1.3 | 1.2 |
| 70–74 | 100 | 1.4 | 1.3 |
| ≥75 | 62 | 1.6* | 1.2 |
| | | trend $\chi_1^2=4.88$ $P=0.03$ | trend $\chi_1^2=0.5$ $P=0.48$ |

*$P<0.05$.
[a]Ratio of death rates in excess of that expected from natural mortality.

*This paper was written by A. Babiker, R.J. Shearer and C.E.D. Chilvers on behalf of the Co-operative Urological Cancer Group. Members of the group are listed in the achnowledgements.
Correspondence: R.J. Shearer, The Royal Marsden Hospital,Fulham Road, London SW3 6JJ, UK.
Received 3 November 1988.

mortality revealed no significant effect of age ($\chi^2=1.97$, d.f. $=1$, $P=0.16$) or sex ($\chi^2=1.18$, $P=0.27$). Thus there is no evidence of any intrinsic prognostic effect of these two demographic factors.

The performances of the other variables considered separately (but each adjusted for age and sex) are shown in Table II. The factor with the greatest predictive power (as measured by the value of $\chi^2$) was primary tumour size (trend $\chi^2=9.94$, d.f. $=1$, $P=0.002$). This is illustrated graphically in Figure 1a. Median survival times for small, moderate and large tumours were 36, 24 and 15 months respectively. The corresponding 5-year survival probabilities were 34, 28 and 23% respectively.

**Table II** Prognostic value of non-demographic factors

| *Factors and levels* | *Number of patients* | *Rate ratio*[a] |
|---|---|---|
| *Tumour size* | | |
| Small | 42 | 1.0 |
| Moderate | 165 | 1.5 |
| Large | 155 | 1.9** |
| | | Trend $\chi_1^2=9.94$ $P=0.002$ |
| *Nodal status* | | |
| Node-negative | 129 | 1.0 |
| Node-positive | 40 | 1.7* |
| *Tumour appearance* | | |
| Papillary | 37 | 1.0 |
| Solid | 340 | 1.3 |
| *Histology* | | |
| Transitional cell | 325 | 1.0 |
| Squamous cell | 11 | 1.7 |

*$P<0.05$; **$P<0.01$.
[a]Adjusted for age and sex.

The second most predictive variable was nodal status ($\chi^2=5.41$, d.f. $=1$, $P=0.02$). The estimated median survival time and 5-year survival probability for node-positive patients were 14 months and 18% compared to 30 months and 33% for node-negative patients. Figure 1b gives the survival curves for the two groups.

The data do not provide evidence of a significant effect of tumour appearance and cell type on prognosis. The lack of evidence for the latter variable may be due to the small number of patients with squamous cell carcinoma.

Investigation of the joint prognostic effect of the above variables revealed a very significant interaction between tumour size and nodal status ($\chi^2=10.50$, d.f. $=2$, $P=0.005$). Table III gives the mortality rate ratios for the combinations of the levels of these two factors. The rate ratios are relative to the node-negative small tumour category. The positive and highly significant trend in mortality rate with tumour size ($\chi^2=12.70$, d.f. $=1$, $P<0.0005$) when there is no involvement of the local lymph nodes is contrasted with an apparent negative trend among the node-positive patients. However, this negative trend in the latter group is not statistically significant ($\chi^2=2.42$, d.f. $=1$, $P>0.1$). Further analysis of this interaction showed that more than 90% of its effect (as measured by $\chi^2=10.50$, d.f. $=2$) was due to the difference in the prognostic effect of nodal status within patients with large tumours on the one hand, and small or moderate tumours on the other hand. Furthermore, there was no significant difference in mortality rate between node-positive and node-negative patients with large tumours. This suggested that primary tumour size and nodal status might

**Figure 1** (a) Survival by size of primary tumour: ——— <2.5 cm max. diameter; ------ 2.5–4.4 cm max. diameter; ····· ≥4.5 cm max. diameter. (b) Survival by nodal status: ——— node-negative; ------ node-positive. (c) Survival by tumour appearance: ——— solid; ------ papillary. (d) Survival by histological type: ——— squamous cell; ------ transitional cell.

**Table III** Death rate ratios according to tumour size and lymph nodes involvement

| | Tumour size | | |
|---|---|---|---|
| *Nodal status* | *<2.5 cm* | *2.5–3.4 cm* | *⩾4.5 cm* |
| Node-negative | 1.0 | 2.1 | 3.9** |
| Node-positive | 5.5* | 5.4** | 2.7 |

*$P<0.05$; **$P<0.01$.

**Table IV** Mortality according to prognostic group

| *Group*[a] | *No. patients* | *Mortality rate ratio*[b] |
|---|---|---|
| Group 1 | 14 | 1.0 |
| Group 2 | 66 | 2.1 |
| Group 3 | 82 | 4.0** |

**$P<0.01$.

[a]See text for definition; [b]Trend $\chi^2=16.4$, d.f. = 1, $P<0.0001$.

**Figure 2** Survival by prognostic group: ——— group 1, node-negative and small tumour; ------ group 2, node-negative and moderate tumour; ······ group 3, node-positive and/or large tumour.

be combined into the following prognostic groupings: (1) node-negative and small tumour; (2) node-negative and moderate tumour; (3) node-positive or large tumour. The mortality rate ratios for these groups are given in Table IV and the corresponding survival curves in Figure 2. The mortality rate is approximately doubled from one level to the next. The predictive power is measured by the highly significant trend ($\chi^2=16.40$, d.f. = 1, $P<0.0001$).

### Discussion

Conflicting results have been reported on the prognostic effect of age and gender (Bloom *et al.*, 1982; Narayana *et al.*, 1983; Pryor, 1973; Blandy *et al.*, 1980). In terms of (unadjusted) survival, the present series showed a significant negative trend in survival rate with age (younger patients faring better than older ones) and weak evidence of poorer prognosis for men as compared to women. However, after correcting for natural mortality, the adjusted rates showed no significant dependence on age or sex. The material influence of natural mortality is due to the old age of the patients entered and the length of the follow-up period. Our findings highlight the need for taking natural mortality into account when investigating prognostic factors in trials of moderate length with elderly patients. A simple way of achieving this is to adjust the analysis for age and sex even if the influence of age and sex is not statistically significant, Alternatively, the analysis could be based on mortality specific to the disease under study. This can be more efficient if the disease specific mortality is much lower than natural mortality from all causes, but it is of less value when the recorded cause of death is not very accurate, as is sometimes the case, particularly in elderly patients and in situations when death from other causes may be indirectly related to the study disease or its treatment. In the present series 202 patients have died from bladder cancer, seven from post-operative complications and 51 from other causes. The number of deaths from causes other than bladder cancer or operative mortality was significantly higher than the total number expected (36.5) from all cause natural mortality ($P=0.01$), suggesting, as expected, that these deaths were bladder cancer related.

Invasive papillary carcinomas are reported to be more radiosensitive, less likely to spread to the pelvic nodes and confer more favourable prognosis than invasive solid tumours (Slack & Prout, 1980; Heney *et al.*, 1983). In the present series, nodal status was recorded for 17 patients with papillary tumours, of whom five (23%) were node positive. The corresponding figure for 109 patients with solid tumours and ascertained nodal status was 34 (24%), providing no evidence of a difference in the tendency to metastasise locally. Similarly there was no overall survival difference in the two groups (see Figure 1c).

Although the mortality rate in the few patients with squamous cell tumours was 70% higher than in those with transitional cell tumours, there is little evidence of a real survival difference Figure 1d). This may be due to the small number of patients in the former group (11 patients with squamous cell carcinoma versus 325 with transitional cell carcinoma).

The poor performance of large primary tumours has been reported by Narayana *et al.* (1983) and, at least within one treatment arm, by Bloom *et al.* (1982). We found that the presentation size of the primary tumour was the single most important prognostic factor. The mortality rates for small, moderate and large tumours were, on average, in the ratios 1:1.5:2.

Metastasis to the regional lymph nodes is well recognised as an indicator of poor prognosis. Smith & Whitmore (1981) reported 7% 5-year survival in 134 node-positive patients. Heney *et al.* (1983) found 3% 5-year survival in 23 node-positive patients compared to 41% in 59 node-negative patients, while Bloom *et al.* (1982) reported 18 and 53% (corrected) 5-year survival for the two groups. Our data confirm these findings. We found nodal status to be the second most important prognostic variable. On average, node-positive patients experienced a 70% higher mortality rate than node-negative patients.

Primary tumour size and nodal status do not appear to act independently. We found a very significant interaction between the effects of these two variables on prognosis. A highly significant positive trend in mortality with tumour size in node-negative patients is in contrast to a negative though not quite significant trend in node-positive patients. The fact that the latter is not statistically significant suggests that the nature of this interaction is likely to be quantitative rather than qualitative, where by a 'quantitative interaction' between two factors A and B, we mean a situation where there is variation in magnitude, but not in direction, of the effect of A among the levels defined by B and vice-versa. The situation where there is reversal of direction is termed qualitative or cross-over interaction. One possible explanation for the lack of any difference in mortality rate between node-positive and node-negative patients with large tumours is that involvement of the lymph nodes tends to be underdetected in general, and that large tumours are much more likely to have metastasised to the local lymph nodes than small or moderate tumours.

Based on the above findings, we propose the following prognostic groupings: (1) node-negative and small primary tumour; (2) node-negative and moderate primary tumour; (3)

node-positive or large primary tumour. This grouping accounted for more than 75% of all variation in survival accounted for by all the six variables and their first order interactions. However, the true performance of this grouping can only be judged on independent data.

This work was supported in part by the Cancer Research Campaign and the Medical Research Council (A.B., J.M.B., C.E.D.C. and E.M.W.). Members of the group are listed below.
G.F. Abercrombie, St Mary's Hospital, Portsmouth PO3 6AD; A. Babiker, Institute of Cancer Research, Sutton SM2 5NG; J.M. Bliss, Institute of Cancer Research, Sutton SM2 5NG; H.J.G. Bloom, The Royal Marsden Hospital, London SW3 6JJ; P.J.R. Boyd, St Helier Hospital, Sutton SM5 1AA; C.H.T.D. Brown, Peterborough District Hospital, Peterborough PE3 6DA; R.B. Buchanan, Wessex Regional Radiotherapy Centre, Southampton SO9 4HA; A.H. Calvert, The Royal Marsden Hospital, Surrey SM2 5PT; R.K. Carruthers, Kent and Canterbury Hospital, Canterbury CT1 3NG; C.E.D. Chilvers, Institute of Cancer Research, Sutton SM2 5NG; M. Claridge, Kent and Canterbury Hospital, Canterbury CT1 3NG; G.P. Deutsch, Royal Sussex County Hospital, Brighton BN2 5BE; K.R. Durrant, The Churchill Hospital, Oxford OX3 7LJ; L.E. Edwards, St Stephen's Hospital, London SW10; D.P. Fawcett, Battle Hospital, Reading RG3 1AG; G.J. Fellows, Radcliffe Infirmary, Oxford OX2 6HE; H.T. Ford, The Royal Marsden Hospital, Surrey SM2 5PT; E.M. Gordon, St George's Hospital, London SW17 0RE; S. Harland, St Peter's Hospital, London WC2; N.W. Harrison, Hove General Hospital, Hove BN3 3HG; W.E.F. Hendry, The Royal Marsden Hospital, London SW3 6JJ; N.J. Hodson, Royal Sussex County Hospital, Brighton BN2 5BE; A. Horwich, The Royal Marsden Hospital, Surrey SM2 5PT; N. Howard, Charing Cross Hospital, London W6 9RF; J.D. Jenkins, Southampton General Hospital, Southampton S09 4XY; M.A. Jones, Sandwell District General Hospital, West Bromwich B71 4HJ; J.S. Malpas, St Bartholomew's Hospital, London EC1; A.D. Mee, Northwick Park Hospital, Harrow; E. Newlands, Charing Cross Hospital, London W6 9RF; R.T.D. Oliver, London Hospital, Whitechapel, London E1 1BB; R.H. Phillips, St Stephen's Hospital, London SW10; P.N. Plowman, St Bartholomew's Hospital, London EC1; P.R. Riddle, Institute of Urology, London WC2; M.G. Royle, Hove General Hospital, Hove BN3 3HG; R.J. Shearer, The Royal Marsden Hospital, London SW3 6JJ; C.J. Smart, Southampton General Hospital, Southampton SO9 4XY; I.E. Smith, The Royal Marsden Hospital, Surrey SM2 5PT; P.A. Trott, The Royal Marsden Hospital, London SW3 6JJ; A.G. Turner, Peterborough District Hospital, Peterborough PE3 6DA; J. Vinnecombe, St Mary's Hospital, Portsmouth PO3 6AD; H.N. Whitfield, St Bartholomew's Hospital, London EC1; E.M. Williams, Institute of Cancer Research, Sutton SM2 5NG; G. Williams, Hammersmith Hospital, London W12 0HS; G.B. Williams, Charing Cross Hospital, London W6 9RF; C.R.J. Woodhouse, The Royal Marsden Hospital, London SW3 6JJ.

### References

BLANDY, J.P., ENGLAND, H.R., EVANS, S.J.W. & 6 others (1980). T3 bladder cancer – the case for salvage cystectomy. *Br. J. Urol.*, **52**, 506.

BLOOM, H.J.G., HENDRY, W.F., WALLACE, D.M. & SKEET, R.G. (1982). Treatment of T3 bladder cancer: controlled trial of preoperative radiotherapy and radical cystectomy versus radical radiotherapy. *Br. J. Urol.*, **54**, 136.

SHEARE, R.J., CHILVERS, C.E.D., BLOOM, H.J.G., BLISS, J.M., HORWICH, A. & BABIKER, A. for Co-operative Urological Cancer Group (1988). Adjuvant chemotherapy in T3 carcinoma of the bladder; a prospective trial. A preliminary report. *Br. J. Urol.*, **62**, 558.

COX, D.R. (1972). Regression models and life tables. *J. R. Stat. Soc. B*, **34**, 187.

HENEY, N.M., PROPPE, K., PROUT, G.R. JR., GRIFFIN, P.P. & SHIPLEY, W.U. (1983). Invasive bladder cancer: tumour configuration, lymphatic invasion and survival. *J. Urol.*, **130**, 895.

KAPLAN, E.L. & MEIER, P. (1958). Non-parametric estimation from incomplete observations. *J. Am. Stat. Soc.*, **53**, 457.

NARAYANA, A.S., LOENING, S.A., SLYMEN, D.J. & CULP, D.A. (1983). Bladder cancer: factors affecting survival. *J. Urol.*, **130**, 56.

OFFICE OF POPULATION CENSUSES AND SURVEYS (1985). *Mortality Statistics Cause 1984*. Series DH2 No. 11. Her Majesty's Stationery Office: London.

PRYOR, J.P. (1973). Factors influencing the survival of patients with transitional cell carcinoma of the urinary bladder. *Br. J. Urol.*, **45**, 586.

SLACK, N.H. & PROUT, G.R. (1980). The heterogeneity of invasive bladder carcinoma and different responses to treatment. *J. Urol.*, **123**, 644.

SMITH, J.A. JR. & WHITMORE, W.F. JR. (1981). Regional lymph node metastasis from bladder cancer. *J. Urol.*, **126**, 591.